# Cultural Differences in Stress-Related Psychological, Nutrition, Physical Activity and Oral Health Factors of Professors

**DOI:** 10.3390/nu12123644

**Published:** 2020-11-27

**Authors:** Laura Redondo-Flórez, Jesús Fernández-Lucas, Vicente Javier Clemente-Suárez

**Affiliations:** 1 Faculty of Sports Sciences, Universidad Europea de Madrid, 28670 Madrid, Spain; lauraredondo_1@hotmail.com; 2 Applied Biotechnology Group, Biomedical Science School, Universidad Europea de Madrid, 28670 Villaviciosa de Odón, Spain; jesus.fernandez2@universidadeuropea.es; 3 Grupo de Investigación en Ciencias Naturales y Exactas, GICNEX, Universidad de la Costa, CUC, Barranquilla 080002, Colombia; 4 Grupo de Investigación en Cultura, Educación y Sociedad, Universidad de la Costa, Barranquilla 080002, Colombia

**Keywords:** stress, burnout, professors, physical activity, nutrition, oral health

## Abstract

With the aim to explore cultural differences in stress-related psychological, nutrition, physical activity, and oral health factors between Spanish and Latin American professors, we analysed stress-related factors in 598 professors (39.9% male, 60.1% female, 41.3 ± 9.8 years) by a collection of questionnaires, which involved psychological, nutritional, physical activity and oral health items. Results showed how Spanish professors presented significantly (*p* ≤ 0.05) higher scores than Latin American professors in perceived stress (Spanish: 21.40 ± 4.32 vs. Latin American: 20.36 ± 4.31), teaching stress (Spanish: 6.59 ± 2.28 vs. Latin American: 6.00 ± 2.99) and neuroticism (Spanish: 5.40 ± 2.10 vs. Latin American: 4.58 ± 1.72). Spanish professors also showed healthier nutritional and physical activity habits than their Latin American counterparts, presenting higher consumption of milk products and a higher numbers of meals per day, greater weekly meat and fish consumption and higher weekly resistance training, as well as less eating between hours and snacking consumption. Nevertheless, Spanish professors brushed their teeth less and showed a higher smoking habit than Latin American professors. We concluded that there were cultural differences between Spanish and Latin American professors. In the present research, Spanish professors showed significantly higher burnout levels, teaching stress, perceived stress, and neuroticism than Latin American professors, and several differences were also found around health behaviours. These differences in perceived stress, teaching stress and burnout syndrome may be due to the habituation process of Latin American professors, and probably are associated with a higher stressful and demanding socio-cultural context.

## 1. Introduction

Stress response is one of the older mechanisms that human beings developed to be able to adapt to the demands of changing and highly eliciting contexts [1]. Stressors could be either external (from the context/environment, psychological or social situations) or internal (illness, regeneration, recovery and return to homeostasis processes). Stress produces no specific response, which triggers neurologic, endocrinologic, psychological, cognitive and behavioural modifications, mostly regulated by the hypothalamus-pituitary-adrenal gland (HPA) axis and the autonomous nervous system by a negative feedback [1,2] whose aim is the maintenance of organic homeostasis through metabolic, cardiovascular, immunological and sympathovagal control [3]. After the exposure of a stressor stimuli, the HPA axis is activated and produces an increase of the heart rate, blood pressure, glucose levels, bronchodilation, as well as rises in cognitive and metabolic resources [3,4,5].

Several authors have connected repeated stress exposition with different health problems which may lead in psychopathologies such as anxiety, depression and posttraumatic stress disorder [6,7,8]. Specifically, in work environments the burnout syndrome is considered as a result of uncontrolled stress [9]. In line with this, the academic university scenario has been found to be a stressful context for both students [10,11,12,13,14,15] and professors [16], since both presented a hyperactivation of the autonomous sympathetic nervous system. Therefore, the educational context was previously considered as a focus of sustained stress for teachers, since the burnout syndrome was a common pathology of this professional collective [17]. Professors may thus feel emotionally exhausted and constantly tired, probably due to associated sleep problems. In the psychosomatic field, they may suffer frequent headaches, gastrointestinal disorders, as well as modification in appetite and food ingestion patterns. Regarding the emotional and behavioural symptoms, they may develop low tolerance to frustration and poor personal fulfilment, irritability, impatience and depression [18]. Furthermore, previous studies linked professional stress with the appearance of physical and mental diseases, being related to approximately 50% of medical leaves [18].

Diverse factors have highlighted the capability of subjects to modulate the stress response. In line with this, nutrition is considered as one of these key factors that could modulate stress response. Previous studies described how university students with a non-healthy diet (constituted by high carbohydrates, fast food and saturated fats consumption) was related with greater episodes of depressive states, anxiety and perceived stress [19,20,21,22]. Furthermore, previous literature described how stress altered eating behaviour, showing an increase in calories consumption [23,24,25]. Recent studies, in contrast, suggest that healthy eating is not directly associated with stress reduction since authors described that healthy eating behaviour might not be directly related to an instant decrease in stress but it might constitute a useful tool in the long term [26,27]. Another important factor in stress response modulation is physical activity. Previous studies found the benefits of physical activity since regular exercise practice has been associated with significant lower perceived stress levels [26,28,29] as a consequence of the increase of neurotransmitters (dopamine, serotonin and endorphins) during and after the exercise, which are responsible for the autonomous nervous system modulation [30,31,32]. For example, mind–body activities such as yoga and qigong have been highlighted as useful tools which could contribute to stress management [33]. Finally, the oral health status was also defined as a stress modulator since greater levels of stress have been related with alterations in oral health behaviour and oral health care, a fact that may be important in susceptible people which could develop periodontal disease [34]. Additionally, positive associations were found between stress and the presence of plaque and gingivitis [35], as well as oral functional problems, such as chewing and speaking difficulties, which have been associated with mental health [36].

Another important factor related to stress is the cultural factor. Based on the current data of the United Nations on Drugs and Crime’s International Homicide Statistics, Latin America and the Caribbean display the highest levels of international homicides in America, with 23 per thousand people in 2015, while Euro zone has been underlined as one of the regions with the lowest rates, with 1 per thousand people in 2015. These cultural differences in aggressive social behaviours and violence present a direct effect on stress related factors as the autonomic modulation, showing that Latin America university students have higher sympathetic modulation during their clinical stay than European students [13]. Other authors consider socio-cultural Latin American conditions as stimuli that would produce higher levels of stress due to the eliciting social environment and violent context [37,38,39,40].

Considering the multifactorial basis of stress and the cultural effect in stress management to prevent work-related problems such as burnout syndrome, herein we report an in-depth analysis of cultural differences in stress-related psychological, nutrition, physical activity and oral health factors in Spanish and Latin American professors. As a result, we hypothesized that Spanish professors would present lower burnout levels and different psychological, nutrition, odontological and physical activity behaviours than Latin American professors. 

## 2. Materials and Methods

### 2.1. Participants

598 higher education professors were analysed; 39.9% were male and 60.1% were female, their mean age was 41.3 ± 9.8 years, and 80.4% were Spanish and 19.6% were Latin Americans (Mexico, Colombia, and Ecuador). All of them lived in the same country in which they were working. Their specialization areas were Sciences, Health Sciences, Art and Humanities, Engineering and Architecture and Social and Legal Sciences (Table 1). Participants were enrolled through a volunteering call that was made to the research centres of each University. All the procedures were conducted following the Helsinki Declaration (as revised in Brazil, 2013), and approved by the University Ethic Committee (CIPI/18/074). Informed consents were also signed before the start of the research.

### 2.2. Design and Procedure

Cultural differences in psychological profile, nutrition habits, physical activity habits and oral health status were analysed by a compendium of questionnaires, as follows.

#### 2.2.1. Anthropometric Measures

Height and Weight. Participants took measurements themselves and entered the results into the questionnaires. These variables were considered for body mass index (BMI) analysis.

#### 2.2.2. Teaching Activity Measures

Teaching experience was measured on a free choice scale, indicating the years of professional experience in the teaching service.

Teaching Satisfaction. This item was measured on a Likert scale from 0 to 10, where 0 = Not all satisfied and 10 = Completely satisfied, to indicate the professor’s satisfaction in their job, considering their current situation.

Teaching Stress. As in the previous item, this item was measured on a Likert scale from 0 to 10, where 0 = None and 10 = Severe, to indicate the current stress perception.

#### 2.2.3. Psychological Measures

Psychological measures were performed by the following questionnaires:

Maslach Burnout Inventory Test [41]. This test assesses the presence of burnout syndrome. It is composed of 22 items that are answered in a six-point Likert scale, where 0 = Never and 6 = Daily. An example item is: “I worry that this job is hardening me emotionally”.

Perceives Stress Scale (PSS) [42]. This scale assesses the level of perceived stress in a one-month period. It is composed of 14 items answered in a five-point Likert scale, meaning 0 = Never and 4 = Very often. An example item is: “In the last month, how often have you been able to control irritations in your life?”

NEO Five-Factor Inventory (NEO-FFI) [43]. This scale analyses five factors of personality: neuroticism, extraversion, openness, kindness and responsibility. In our present research we used a condensed version which included ten items, each answered by a five-point Likert scale, where 0 = Strongly disagree and 4 = Strongly agree. An example item is: “I see myself as a person who gets nervous easily”.

Acceptance and Action Questionnaire AAQ-II [44]. This test analyses the psychological inflexibility or experiential avoidance through 7 items, each answered by a seven-point Likert scale, where 0 = Never true and 7 = Always true. An example item is: “Emotions cause problems in my life”. High scores suggest that it is probable that there is current clinical distress.

UCLA Loneliness Scale [45]. This scale assesses the measurement of loneliness. In the present study we used a condensed version composed by three items, each answered by a three-point Likert scale, where 1 = Never and 3 = Frequently. An example item is: “My interests and ideas are not shared by those around me”.

#### 2.2.4. Physical Activity Measures

This variable was analysed by a questionnaire using by previous authors [46] which includes the following items: “How many hours are you physically active, in movement, per day?”; “Do you practise any endurance-based exercise (swimming, cycling, running…)? If yes, how long do you train per week?”; “Do you practise any sport team related exercise (football, rugby, hockey…)? If yes, how long do you train per week?” “Do you practise any strength-based sport (weights, CrossFit, calisthenics …). If yes, how long do you train per week?”. Questions should be answered by choosing the option offered which fits the best with the physical activity developed by the subject. According to the first question, “How many hours are you active, in movement, per day?” answers ranged from “less than 30 minutes” to “more than three hours”. In the case of the rest of questions, the answers range from “I do not practise this kind of training” to “more than 12 hours”.

#### 2.2.5. Nutrition Habits Measures

We adapted previous used questionnaires [46,47,48] to analyse eating habits and nutrition behaviours in the population analysed in the present research. We used two groups of questions; the first group concerned nutrition behaviours and perceptions (5 questions): “Do you have the self-perception that you are consuming healthy food?”, “Do you eat between hours?”, “Are you following a diet?”, “Do you read food labels?”, “Do you eat slowly?”. In these five items, participants give a response with dichotomous answers, where 0 = No and 1 = Yes. The rest of the questions were related to weekly consumption frequency (ranged from 1 to “more than 5”) of different food groups, including meat, fish, legumes, fast food, snacks, fried foods, fermented or soft drinks, as well as daily consumption frequency of milk products and water and the number of meals made per day. Questions relating to daily hours of sleep were also included in this section.

#### 2.2.6. Oral Health Measures

Oral health was measured following a previously used questionnaire [46,47] consisting of six items related to oral health; the first four of them were answered by “yes, sometimes or no”. The rest of the questions included “How many times a day do you brush your teeth?”, for which answers ranged from “none” to “more than four”, and “Do you smoke?”, for which answers ranged from “no” to “more than five cigarettes per day”.

### 2.3. Statistical Analysis

Statistical analyses were analysed using the Statistical Package for the Social Sciences (SPSS) version 24.0 (SPSS Inc., Chicago, Ill., USA). Descriptive statistics (mean and standard deviation) were calculated for each variable. Kolmogorov–Smirnov tests were performed to analyse normality and homogeneity of each variable. A MANOVA with nationality as the fixed factor was conducted to explore differences between professors for the variables analysed. The level of significance was set at *p* ≤ 0.05. F represents the F value and η^2^ represents partial eta square.

## 3. Results

Data are presented as mean ± standard deviation. The MANOVA presented significant differences between the two groups analysed: F = 9.913; *p* = 0.000; η^2^ = 0.464. Spanish professors showed significantly higher teaching stress, perceived stress, extraversion, agreeableness, neuroticism, and hours of sleep while having lower values of personal fulfilment and openness to experience than Latin American professors (Table 2).

Spanish professors showed significantly higher height values and lower values of weight and body mass index than the Latin American professors.

Spanish professors presented significantly higher values in the number of meals and milk products per day, meat and fish consumption per week and weekly resistance training than Latin American professors. However, they showed lower values in eating between hours, following a diet, and legume, soft drink, and snack consumption per week than Latin American professors (Table 3).

**Figure 1:**
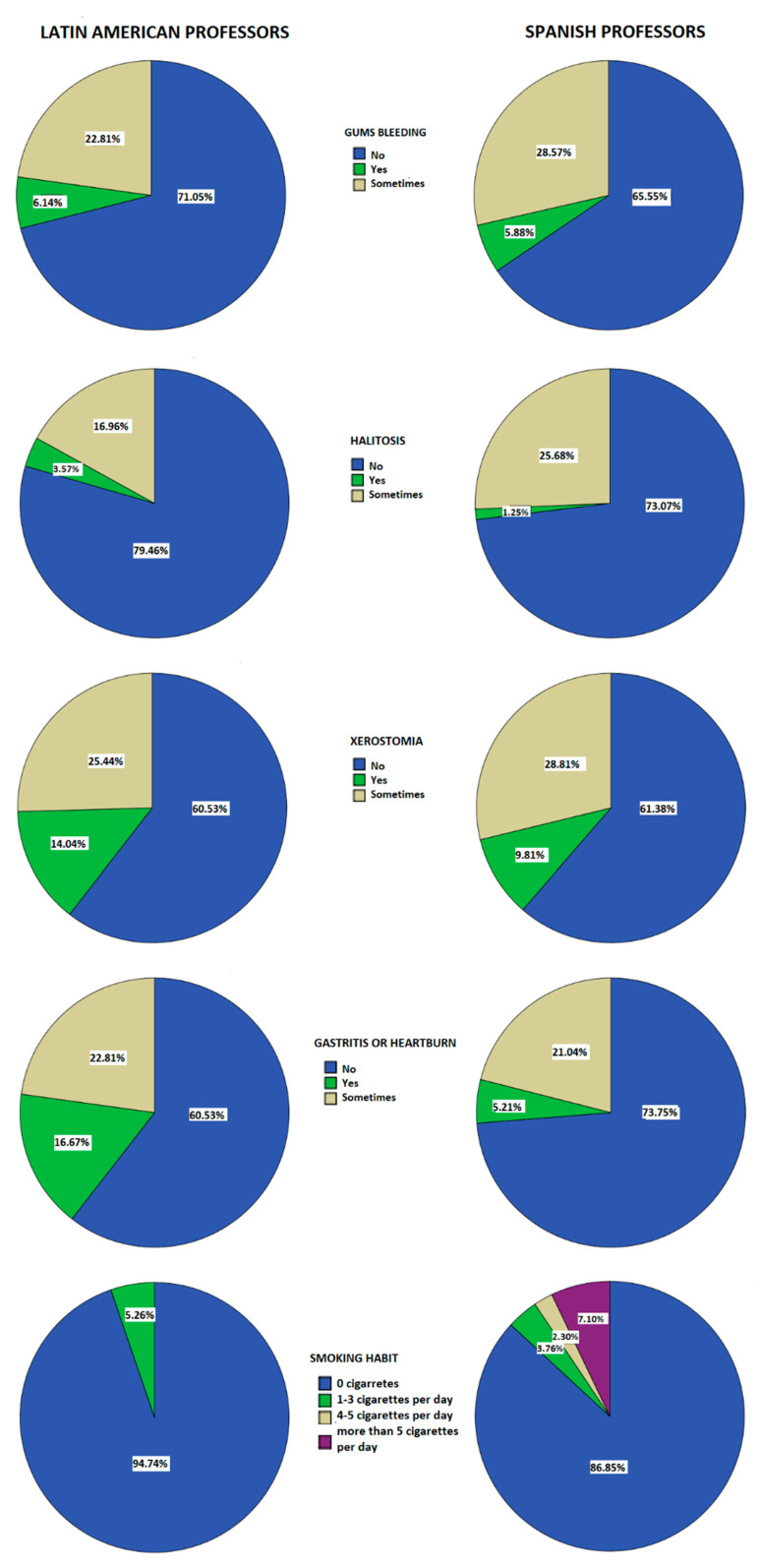
Oral health variables analysed.

Regarding oral health, Latin American professors showed significantly higher values in daily toothbrushing than Spanish professors (F = 15.717, *p* = 0.00, η^2^ = 0.027) and significantly lower values in a smoking habit (F = 11.69, *p* = 0.001, η^2^ = 0.02) (Figure 1).

## 4. Discussion

The aim of the present study was to analyse cultural differences in stress-related psychological, nutrition, physical activity, and oral health factors of Spanish and Latin American professors. Since Spanish professors showed higher burnout levels, as well as different psychological, nutrition, odontological and physical activity behaviours, than Latin American professors, the initial hypothesis was partially confirmed. 

In the present study, Spanish professors presented higher levels of teaching stress and perceived stress than Latin American counterparts. Additionally, Spanish professors showed greater extraversion, agreeableness, and neuroticism values than Latin American professors. Previous researchers found how high scores on these psychological constructs were related with difficulties, including but not limiting emotional regulation, maladaptive behaviours and psychopathologies, such as anxiety or depression [43,48,49,50], highlighting neuroticism as the main characteristic of burnout [51]. Moreover, several authors described how a maintained stress exposition may exacerbate these psychopathologies, including depressive states, anxiety and post-traumatic stress disorder; this fact may be considered a problem since it could trigger chronic mental diseases [7,8,52]. Furthermore, our results regarding Spanish professors agree with previous findings reported by a national survey conducted in Spain in 2010, showing a no positive evolution of these collectively [53]. Data obtained were also in consonance with Norway, another eurozone country, where professors experienced high degrees of stress and burnout syndrome in their work environment [54]. Nevertheless, Latin American professors showed lower perceived stress and burnout levels, which could be explained by the habituation process they may suffer in response to their higher stressful and highly demanding socio-cultural environment. Regarding personal fulfilment, the low scores presented in Spanish professors were also consistent with the findings of previous researchers when analysing burnout syndrome in a large variety of health and service occupations including professors, nurses, social workers, psychologists and police [55,56,57].

According to dietary habits, several differences were found between both groups. Spanish professors presented significantly higher values in the number of meals and lower values in eating between hours than Latin American professors, results which were consequent with previous dietary guidelines, following the recommendation of making 5 meals per day and avoiding eating between hours [58]. Furthermore, Spanish professors showed a significant greater consumption of milk products per day, as well as higher meat and fish consumption per week (following the dietary recommendations included in the Spanish program AESAN NAOS Strategy [59]), than Latin American professors. In this line, pre and probiotics presented in milk products altered gut microbiota composition due to their capability to improve the growth of favourable bacteria in the gut [60]. Moreover, dairy pre and probiotic products intake have been highlighted by previous researchers as a powerful HPA axis modulator, as well as a gut modulator, being related to psychological disorders, such as anxiety and depression [61,62,63]. Then, since their milk products consumption is significantly higher, Spanish participants could be more benefited from this protective effect of pre and probiotics in stress modulation than Latin American professors. In contrast, Latin American professors showed unhealthier dietary habits since they consumed more soft drink and snacks per week than the Spanish professors, although they also showed higher legume consumption per week than Spanish professors. This higher snack consumption in Latin American professors could be explained by the fact that daily worries which they may suffer, probably due to their unstable social environment, could increase unhealthy eating behaviour, with a tendency towards high fat and high sugar snacking [64], or because they ate fewer main meals during the day and the intervals between meals were longer, so they felt more hungry. Similar results were also found in previous studies where an inverse relationship between affect changes, including a better feeling and a reduction in the stress levels after the consumption of carbohydrates was found [65,66,67]. Furthermore, results relating to dietary habits showed by Latin American professors were also supported by their significantly higher values in body mass index, probably due to their worse dietary habits and low sports practise, showing significantly lower rates in weekly resistance training than the Spanish professors. Thus, considering these results we could say Spanish professors had healthier dietary habits than Latin American ones.

Relating to oral health, Latin American professors presented significantly higher values in daily toothbrushing than Spanish professors. This high frequency in toothbrushing could be related to high neuroticism levels [68]. However, our results were not consistent with these findings since Latin American professors presented significantly lower neuroticism values. Furthermore, no significant associations were found between toothbrushing and psychological factors [69]. Nevertheless, previous researchers pointed out a relation between stress and oral health, showing how there is an association between academic or work-related stress and worsening of oral hygiene and increasing gingival inflammation [35,70]. Our results were in consonance with these findings since Spanish professors, who showed higher teaching stress and perceived stress levels, also presented lower toothbrushing frequency. In this line, previous authors highlighted how alterations on the oral microbiome have been related to gut dysbiosis, triggering variations in the gut microbiota’s composition modulating the anxiogenic response [71,72], as well as the strong stress influence in gut microbiota’s composition, generating different gastrointestinal disorders and food allergies due to the modification in motility and visceral sensibility [73,74,75]. Nevertheless, no significant differences in gastrointestinal pathologies (gastritis or heartburn) were found in any group.

Regarding smoking and sleep habits, Spanish professors showed significantly higher values than Latin American professors in both variables. Higher smoking habits presented by Spanish professors who additionally showed higher perceived stress and teaching stress levels, were in consonance with previous researches since it was found how heavy smokers had a higher perceived stress as well as findings that stress raised the necessity to smoke [76,77,78,79]. Nevertheless, sleep habits in Spanish professors were not in consonance with previous literature, since several authors described how stressful periods shortened sleep and produced insomnia [80,81,82].

### 4.1. Limitation of the Study and Future Research Lines

The main limitation of this study was the lack of biological measurement including stress hormones (cortisol, adrenaline, alpha-amylase, etc.), and gut microbial population, which may provide relevant information with the aim to improve the complete understanding of stress related effects in this population. Additionally, economic status was not included in questionnaires and we consider it could be an important issue to take into consideration since an adverse economic situation may have a strong influence on stress. Future studies may address these issues.

### 4.2. Practical Application

The multifactorial analysis in stress-related factors may be considered as a useful tool to measure and prevent stress in professors with the aim to help professors facing stressful situations—helping them develop their skills properly and helping to prevent burnout syndrome.

## 5. Conclusions

There were cultural differences between Spanish and Latin American professors; Spanish professors presented significantly higher burnout levels, teaching stress, perceived stress, neuroticism and different nutrition, physical activity, and oral health pattern than Latin American professors. These differences may be due to the habituation of Latin American professors to their higher stressful and demanding socio-cultural context.

## Figures and Tables

**Table 1:** Gender and specialisation areas distribution in Spanish and Latin American teachers analysed.

Group	Gender		Specialization Areas				
	Male	Female	Science	Health Science	Art and Humanities	Engineering and Architecture	Social and Legal Science
Spanish	38%	62%	10.3%	43.3%	14.1%	9.3%	23.0%
Latin American	51%	49%	14.5%	30.8%	14.5%	6.0%	34.2%

**Table 2:** Teaching activity and psychological variables.

Variable	Spanish (*n* = 481)	Latin American (*n* = 117)	F	*p*	η^2^
Age (years)	41.23 ± 8.77	40.34 ± 13.21	0.714	0.34	0.001
Teaching experience (years)	11.67 ± 8.60	12.74 ± 10.65	1.223	0.27	0.002
Teaching Satisfaction	8.26 ± 1.28	8.41 ± 1.69	0.941	0.33	0.002
Teaching Stress	6.59 ± 2.28	6.00 ± 2.99	4.977	0.02	0.009
Hours of sleep	6.82 ± 0.91	6.46 ± 0.89	12.939	<0.01	0.023
Emotional exhaustion	18.73 ± 10.04	18.63 ± 10.02	0.009	0.92	0.000
Depersonalization	4.09 ± 4.06	3.87 ± 3.77	0.256	0.61	0.000
Personal fulfilment	37.03 ± 5.94	38.50 ± 5.74	5.389	0.02	0.01
Perceived stress	21.40 ± 4.32	20.36 ± 4.31	4.983	0.02	0.009
Psychological Inflexibility	16.03 ± 8.60	14.49 ± 8.15	2.838	0.09	0.005
Loneliness	3.92 ± 1.40	3.86 ± 1.15	0.125	0.72	0.000
Extraversion	5.70 ± 1.81	4.69 ± 1.75	27.411	<0.01	0.047
Agreeableness	7.36 ± 1.48	6.87 ± 1.88	8.715	<0.01	0.015
Conscientiousness	8.18 ± 1.52	1.65 ± 1.08	2.549	0.11	0.005
Neuroticism	5.40 ± 2.10	4.58 ± 1.72	14.171	<0.01	0.025
Openness to experience	7.88 ± 1.75	8.46 ± 1.48	10.29	<0.01	0.018

**Table 3:** Anthropometrical, nutrition and physical activity variables.

Variables	Spanish (*n* = 481)	Latin American (*n* = 117)	F	*p*	η^2^
Height (cm)	170.03 ± 9.82	166.74 ± 8.75	10.087	<0.01	0.018
Weight (kg)	68.57 ± 13.06	73.36 ± 12.47	11.853	<0.01	0.021
Body mass index (kg/m²)	23.75 ± 5.67	26.26 ± 3.27	19.4	<0.01	0.033
Hours of sleep per day	6.82 ± 0.91	6.46 ± 0.89	12.939	<0.01	0.023
Healthy food	0.82 ± 0.38	0.79 ± 0.40	0.516	0.47	0.001
Eat between hours	0.44 ± 0.49	0.69 ± 0.46	22.84	<0.01	0.039
Follow a diet	0.15 ± 0.36	0.32 ± 0.46	16.377	<0.01	0.028
Read food labels	0.62 ± 0.48	0.65 ± 0.47	0.384	0.53	0.001
Slowly eating	0.72 ± 0.44	0.67 ± 0.47	1.27	0.26	0.002
Meals per day	3.93 ± 0.82	3.43 ± 0.91	30.78	<0.01	0.052
Water glasses per day	4.20 ± 1.11	4.17 ± 1.28	0.009	0.92	0.000
Milk products per day	2.16 ± 1.13	1.92 ± 1.52	3.549	0.05	0.006
Snacks per week	1.10 ± 0.90	1.40 ± 1.06	9.835	<0.01	0.017
Meat per week	3.15 ± 1.27	2.36 ± 1.37	32.00	<0.01	0.054
Fish per week	2.30 ± 1.14	1.60 ± 1.14	32.36	<0.01	0.055
Legume per week	2.12 ± 1.14	2.68 ± 1.47	18.60	<0.01	0.032
Fast food per week	0.85 ± 0.89	0.95 ± 1.04	1.021	0.31	0.002
Soft drink per week	1.13 ± 1.57	1.80 ± 1.87	14.314	<0.01	0.025
Fried food per week	1.27 ± 1.20	1.40 ± 1.23	1.061	0.30	0.002
Alcoholic drink per week	0.42 ± 0.65	0.37 ± 0.76	0.446	0.50	0.001
Daily movement (h)	1.53 ± 1.06	1.57 ± 1.12	0.086	0.77	0.000
Weekly strength-based sport (h)	0.68 ± 1.09	0.90 ± 1.39	3.299	0.07	0.006
Weekly team sport (h)	0.23 ± 0.69	0.32 ± 0.83	1.53	0.21	0.003
Weekly resistance training (h)	1.18 ± 1.45	0.84 ± 1.2	5.134	0.02	0.009
